# Single-Cell Molecular and Cellular Architecture of the Mouse Neurohypophysis

**DOI:** 10.1523/ENEURO.0345-19.2019

**Published:** 2020-01-16

**Authors:** Qiyu Chen, Dena Leshkowitz, Janna Blechman, Gil Levkowitz

**Affiliations:** 1 Department of Molecular Cell Biology, Weizmann Institute of Science, Rehovot 7610001, Israel; 2 Bioinformatics Unit, Life Sciences Core Facilities, Weizmann Institute of Science, Rehovot 7610001, Israel

**Keywords:** neuroendocrine, neurohypophysis, oxytocin, pituicyte, pituitary, tanycyte

## Abstract

The neurohypophysis (NH), located at the posterior lobe of the pituitary, is a major neuroendocrine tissue, which mediates osmotic balance, blood pressure, reproduction, and lactation by means of releasing the neurohormones oxytocin (OXT) and arginine-vasopressin (AVP) from the brain into the peripheral blood circulation. The major cellular components of the NH are hypothalamic axonal termini, fenestrated endothelia and pituicytes, the resident astroglia. However, despite the physiological importance of the NH, the exact molecular signature defining neurohypophyseal cell types and in particular the pituicytes, remains unclear. Using single-cell RNA sequencing (scRNA-Seq), we captured seven distinct cell types in the NH and intermediate lobe (IL) of adult male mouse. We revealed novel pituicyte markers showing higher specificity than previously reported. Bioinformatics analysis demonstrated that pituicyte is an astrocytic cell type whose transcriptome resembles that of tanycyte. Single molecule *in situ* hybridization revealed spatial organization of the major cell types implying intercellular communications. We present a comprehensive molecular and cellular characterization of neurohypophyseal cell types serving as a valuable resource for further functional research.

## Significance Statement

The neurohypophysis (NH) is a major neuroendocrine interface, which allows the brain to regulate the function of peripheral organs in response to specific physiologic demands. Despite its importance, a comprehensive molecular description of cell identities in the NH is still lacking. Utilizing single-cell RNA sequencing (scRNA-Seq) technology, we identified the transcriptomes of five major neurohypophyseal cell types in the adult male mice and mapped the spatial distribution of selected cell types *in situ*. We revealed an unexpected cellular heterogeneity of the NH and provide novel molecular markers for neurohypophyseal cell types with higher specificity than previously reported.

## Introduction

The pituitary, also dubbed the hypophysis, is the master endocrine gland that is localized at the base of the hypothalamus in all vertebrate species. It is composed of the adenohypophysis (AH) and the neurohypophysis (NH), also known as the anterior and posterior pituitary, respectively. The mammalian pituitary consists of an additional anatomically discernable tissue, the intermediate lobe (IL), which is located between the NH and AH. However, the IL is not as distinguished in the pituitary of human and some non-mammalian vertebrates, including zebrafish (Gutnick et al., 2011; Norris et al., 2013; Wircer et al., 2016; Larkin and Ansorge, 2017). The hypothalamo-neurohypophyseal system (HNS) encompasses hypothalamic magnocellular neurons residing in the paraventricular nucleus (PVN) and supraoptic nucleus (SON) and project their axons into the NH. Thus, two neuropeptides, oxytocin (OXT) and arginine-vasopressin (AVP), are produced in magnocellular neurons, transported along neurohypophyseal-projecting axons and released into the general blood circulation through the neurohypophyseal capillary plexus (Murphy et al., 2012). Circulating OXT and AVP neurohormones affect the physiologic function of peripheral organs such as the kidney, mammary gland and the uterus. Specifically, AVP regulates osmotic balance and blood pressure (Wircer et al., 2016; Donadon et al., 2018; Olazábal, 2018), while OXT is mainly known due to its effects on reproduction organs (Lee et al., 2009).

Unlike the AH, which serves as a hormone-secreting gland, the NH is a neural tissue, which serves as a neuroendocrine interface between AVP and OXT axonal projections and the permeable capillary network of fenestrated endothelia (Robinson and Verbalis, 2003). This neurovascular interface also contains the pituicytes, specialized neurohypophyseal astroglia, which occupy ∼50% of the neurohypophyseal total volume (Bucy, 1930; Pow et al., 1989). Pituicytes engulf HNS axonal swellings and their terminal buttons and are in close contact with the basal laminar and vascular endothelia (Robinson and Verbalis, 2003; Miyata, 2017). Based on their dynamic morphologic plasticity during lactation and in response to chronic dehydration, it has been suggested that the pituicytes mediate neurohormones passage through the fenestrated capillaries serving as a physical gateway between the axons and the perivascular space (Hatton, 1988; Wittkowski, 1998). Recently, we reported that during development, pituicyte-derived factors regulate the decision of zebrafish NH vasculature to adopt a permeable endothelial fate instead of forming a BBB (Anbalagan et al., 2018). The early definition of pituicytes was based on histochemical staining with silver carbonate and hematoxylin and eosin (Bucy, 1930; Liss, 1958; Dellmann and Sikora, 1981). Thus, different subtypes of pituicytes have been defined by their fibrous, ependymal (with cilia or microvilli), oncocytic morphologies or by ultrastructure of organelle contents, such as dark and pale pituicytes due to high/low density contents of cytoplasmic matrix and organelles and granular pituicytes containing numerous cytosegregosome type dense bodies (Seyama et al., 1980; Wittkowski, 1986; Anbalagan et al., 2018). However, there is very little knowledge of pituicyte-specific genes. Consequently, mammalian pituicytes have been so far labeled with astroglial markers, such as apolipoprotein E (APOE), GFAP, S100β, vimentin (VIM), and connexin43 (Cx43/GJA1), all of which are general astrocytic markers, which are also expressed in other cell types (Cocchia, 1981; Suess and Pliška, 1981; Boyles et al., 1985; Marin et al., 1989; Yamamoto and Nagy, 1993). Moreover, defining and visualizing pituicytes by co-expression of the above genes is not informative as these markers only partially overlap (Wei et al., 2009). Hence, the exact definition of pituicyte cell type and/or subtype remains ambiguous. Finally, other neurohypophyseal cell types might not have been detected in published bulk neurohypophyseal transcriptomic data (Hindmarch et al., 2006).

The recent technological revolution enables high-resolution studies for transcriptome patterns in heterogeneous cell populations. Single-cell RNA sequencing (scRNA-Seq) allows dissecting cell types that are previously hidden due to identical histology, same genetic marker and adjacent location within a complex tissue (Potter, 2018). This technology enables hundreds and thousands of single cells being processed at once, therefore delivers high-throughput, and highly efficient analysis of cell heterogeneity. In this study, we used scRNA-Seq to unravel the cell heterogeneity of the NH. Seven major cell types in the NH and IL of adult male mouse were identified. We present a comprehensive view of the molecular landscape as well as spatial organization of NH and IL cell types, hence providing valuable resources for studying their specific cellular and physiologic functions.

## Materials and Methods

### Experimental design

Three-month-old male C57/BL6 and *Cx3cr1*-GFP mice (Jung et al., 2000) were used in this study. All experimental procedures were approved by the Weizmann Institute’s Institutional Animal Care and Use Committee (IACUC).

### Single-cell dissociation

Two independent groups of five C57/BL6 mice were sacrificed by decapitation and the NH were dissected and collected into ice-cold 1 ml of magnesium-free and calcium-free HBS-/- buffer (20 mM HEPES-buffered saline, 145 mM NaCl, 5.4 mM KCl, and 20 mM glucose, pH 7.2) (Dieck, 1999). NH tissues were then transferred to ice-cold PBS containing magnesium and calcium (HyClone, GE Healthcare), treated with 50 ng/µl Liberase TM (Roche) for 12 min at 37°C, and further dissociated by incubating in HBS-/- buffer containing 0.15 mg/ml Papain (Sigma) and 10 U/ml DNase I (Invitrogen) for 8 min at 37°C. The reaction was stopped by adding heat-inactivated fetal bovine serum (HI-FBS; HyClone) to reach final concentration of 5%. To obtain single-cell resuspension, the loosened tissues were collected and passed through a 40-µm nylon mesh in 800-µl resuspension buffer [Leibovitz L-15 with 0.3 mM glutamine (Gibco, Thermal Fisher), 0.5% of penicillin streptomycin solution (Gibco, Thermo Fisher), 1% HI-FBS, 0.04% BSA]. Cell number, survival rate, clarity, and singularity were checked by Trypan Blue staining followed by hemocytometer counting.

### scRNA-Seq

scRNA-Seq was performed with 10x Genomics Chromium Single Cell kit version 2. Two independent samples, each containing 600–800 cells/ml, which had ∼70% survival rate and very few debris were used to form droplets containing single cell and barcoded-beads. The targeted recovery was 4000 cells per sample. The subsequent cDNA synthesis and library preparations were conducted according to the manufacturer’s protocol (10x Genomics). Two libraries were then indexed and pooled for sequencing using a NextSeq 500 High Output v2 kit (75 cycles; Illumina) according to the manufacturer’s instructions. Four lanes were used with R1 26 cycles and R2 58 cycles.

### Data and software availability

The accession number for the NH single-cell transcriptome reported in this paper is Gene Expression Omnibus (GEO; www.ncbi.nlm.nih.gov/geo): GSE135704.

### Statistical analyses

Sequences data were demultiplexed using Illumina bcl2fastq. Each of the samples was analyzed by Cellranger (version 2.0.0), run with the option –force-cells = 1500 and using the 10X prebuilt mm10 reference database version 1.2.0. The outputs from CellRanger were further analyzed using the Seurat package V2.3 (Butler et al., 2018) and R 3.5. Using Seurat, we performed gene filtering (gene must appear in three cells of a sample) and merging of the cells of both samples to one set. Cell filtering was based on the number of genes per cell (must be between 400 and 5000), the number of UMI counts per cell (between 1000 and 10,000), and the percentage of mitochondria genes lower than 0.25 percentage. Eleven clusters were created with 900 variable genes and 11 principal components (PCs). The cluster names were replaced with the cell type identity based on the differentially expressed genes (marker genes).

### Gene set enrichment analysis

To determine whether known biological functions or gene sets are overrepresented (enriched) in an experimentally-derived gene list, an overrepresentation analysis (ORA) (Boyle et al., 2004) was employed. The gene set associated with a cell type, which was downloaded from PanglaoDB database (Franzen et al., 2019) were compared to the differentially expressed pituicyte markers filtered with criteria of average_logFC ≥ 1 and *p*adj ≤ 0.05. To test for overrepresentation of successes in the sample, the hypergeometric *p* value was calculated using R function phyper with lower tail= false as the probability of randomly drawing k or more successes from the population in n total draws (Kachitvichyanukul and Schmeiser, 1985). The FDR was achieved by adjusting the *p* value using Benjamini and Hochberg (Benjamini and Hochberg, 1995). To further illustrate the above finding specific differentially expressed pituicyte markers were compared with filtering criteria of average_logFC ≥ 1 and *p*adj ≤ 0.05 to published scRNA-Seq gene lists of astrocytes, and tanycytes i.e., PanglaoDB and other studies (Campbell et al., 2017; Chen et al., 2017; Saunders et al., 2018; Zeisel et al., 2018; Franzen et al., 2019).

### Wholemount *in situ* hybridization (WISH) and immunostaining

Three-month-old C57BL6 mice were perfused and fixed by 2% PFA for 10 min and fixed in 4% PFA on ice for 20 min in the dark. WISH was performed as described in (Machluf and Levkowitz, 2011; Wircer et al., 2017) with prolonged proteinase K treatment of 45 min. Tissues were postfixed in 4% PFA for 20 min at room temperature and washed 3 × 15 min PBS-Tx (Triton X-100; 0.3%). Subsequent immunostaining of WISH samples was performed following re-blocking in blocking buffer (10% lamb serum, 0.3% Triton X-100, 1% DMSO in PBS) for 1 h. Primary antibody staining was performed at 4°C overnight. After 3× 30-min PBS-Tx wash, the samples were incubated with 1:200 secondary antibody at 4°C overnight, followed by 3× 30-min PBS-Tx wash and mounting in 75% glycerol. Imaging of WISH samples was performed using Zeiss LSM 800 confocal microscope with oil immersion 40× objective. Whole z-stack maximum intensity projections and cell number quantification of specific cell populations were generated by Fiji-ImageJ software.

### Cryotomy and fluorescent *in situ* hybridization (smFISH)

C57BL6/*Cx3cr1*-GFP transgenic mice were sacrificed by decapitation. The whole pituitary was quickly dissected and fixed in 1% PFA containing 30% sucrose overnight at 4 ^0^C. The fixed tissue was then washed and equilibrated in half Tissue-Tek O.C.T Compound (Sakura) and half 60% sucrose (final 30%) mixture before positioned inside a plastic mold with only O.C.T compound and frozen by burying in dry ice powder. After the whole block turned opaque, it was stored at –80°C in a sealed plastic bag in the dark. Before cryotomy, the embedded O.C.T block was first equilibrated inside the Cryostat machine (Leica) to –25°C for 30 min followed by cryo-sectioning (7 µm) and slice collection on 22 × 22-mm glass coverslips #1 (Thermo Scientific Menzel), precoated with 0.01% L-lysine (Sigma), and stored at –80°C in a Parafilm sealed six-well plate in the dark for up to a month before further digestion and prehybridization steps. smFISH was conducted as described in (Ji and van Oudenaarden, 2012) with the exception that the formamide concentration was increased to 30% for prehybridization and washing. Tissue sections were mounted on Prolong Gold antifade mountant (Thermo Fisher) and images were captured using a wide-field fluorescent microscope (Nikon Eclipse Ti-E) with a cooled CCD camera equipped with oil immersion 60× objective.

### Vibratome sections

Pituitary from *Cx3cr1*-GFP mouse was dissected on ice and fixed in 4% PFA overnight at 4°C. After washing, the pituitary was embedded in 3% Nobel Agarose (BD Biosciences) on ice; 50-µm coronal sections were cut using a Leica VT1000 S vibrating blade microtome (Leica) and then mounted with Aqua-Poly/Mount (Polysciences). The sections were then imaged using a Zeiss LSM 800 confocal microscope.

## Results

### scRNA-Seq revealed seven cell types in the NH and IL

**Figure 1.**
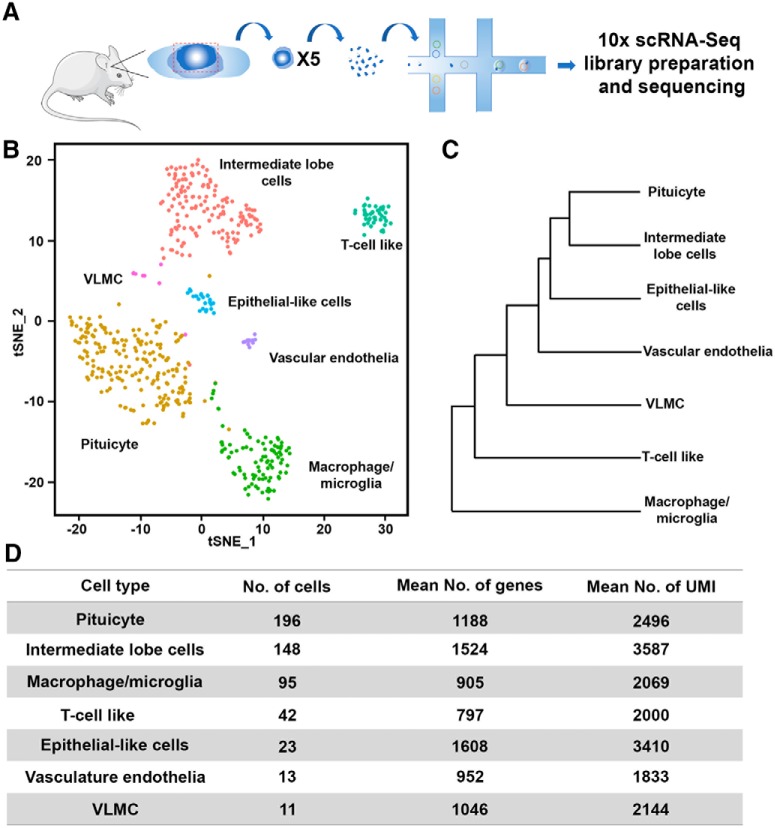
Single-cell RNA-Seq reveals seven cell types of dissected mouse NH. ***A***, Schematic representation of the scRNA-Seq procedure. Neurohypophyseal tissues were dissected from five C57BL6 adult male mice and pooled. Two independent pools were separately subjected to single-cell dissociation, single-cell capturing, and library preparation using the 10x chromium platform. The two libraries were then indexed and combined for sequencing using NextSeq 500 High Output v2 kit (75 cycles). ***B***, The two libraries were pooled and mapped on the tSNE plot, showing cell clusters of IL cells, T-cell like, VLMC, epithelial like cells, vascular endothelia macrophage/microglia, and pituicyte. Each dot represents one cell, and cells with the same color belong to one cell type. ***C***, Dendrogram showing the distance matrix from the PCA space of the average cell among the seven cell types. The length of the path between each two cell types indicates the relativeness between them. ***D***, A table summarizing the number of cells, average number of genes and UMIs found in each cell type.

The pituitary is located within a bony structure of the mouse skull, dubbed sella turcica, allowing accurate surgical isolation of this tissue. In particular, the medially located NH can be readily observed owing to its conspicuous white color, due to the high density of neurohypophyseal axons and pituicytes. We took advantage of these anatomic features to dissect neurohypophyseal tissue from three-month-old C57/BL6 male mice and thereafter performed scRNA-Seq analysis. Notably, the isolated tissue contained residual tissue from the adjacent intermediate pituitary lobe (IL), hence we took into consideration that our NH tissue preparation will contain some IL cells (Fig. 1*A*; Extended Data Fig. 1-1A).

10.1523/ENEURO.0345-19.2019.f1-1 Extended Data Figure 1-1 Adult mouse NH dissection and scRNA-Seq tSNE plot. ***A***, Images showing a dorsal view of the adult male mouse pituitary after the brain has been surgically removed. The left and right images show the pituitary before and after the NH have been dissected. The dashed line indicates the approximate boundary of the NH (scar bars, 50 µm). ***B***, A tSNE plot showing the distribution of individual cells derived from two independent pools of dissected NH in which the cell is colored according to pool origin. Download Figure 1-1, TIF file.

**Figure 2.**
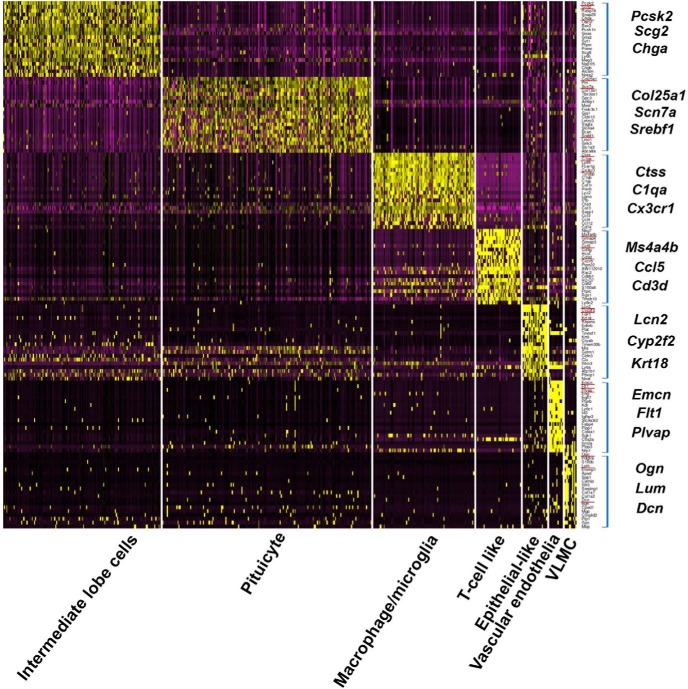
Heatmap of differentially expressed genes in neurohypophyseal and IL cell clusters. Heatmap showing scaled gene expression of the top twenty genes (square brackets) representing each of the seven cell types found in the NH and IL. Each column display gene expression of an individual cell and genes are listed in the rows. Selected marker genes are underlined in red and enlarged on the side.

10.1523/ENEURO.0345-19.2019.f1-2 Extended Data Figure 1-2 Complete list of normalized differentially expressed genes. A complete list of normalized differentially expressed genes that were expressed in at least 25% of the cells within between the two groups of cells. Download Figure 1-2, XLSX file.

**Figure 3.**
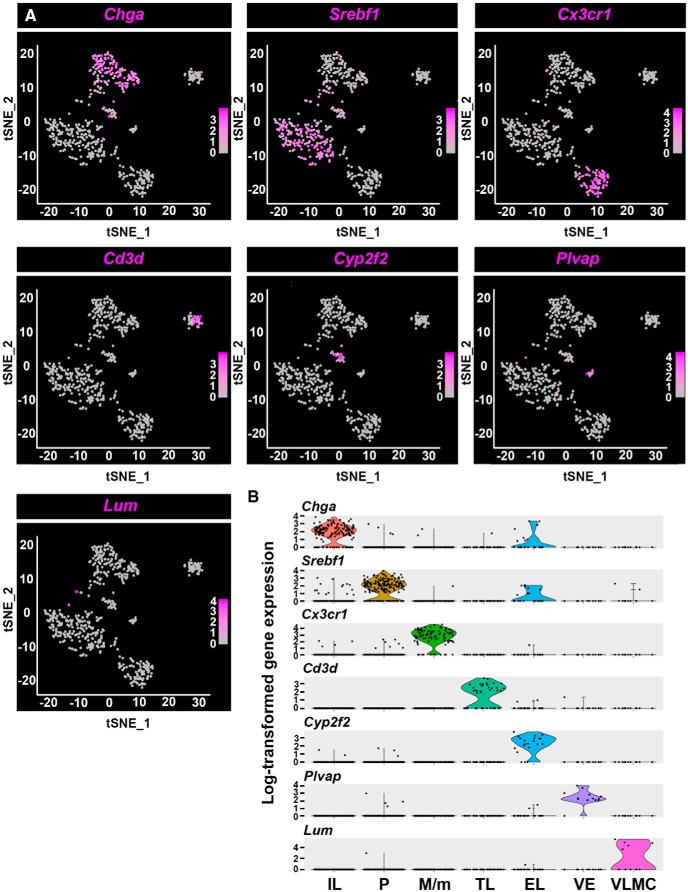
Featured genes representing the landscape of the seven neurohypophyseal and IL cell types. ***A***, Distribution of featured genes from each cell type embedded in tSNE plots. The gene expression scale was color-coded with high expression level in deep blue, low expression in gray. ***B***, Violin plots displaying normalized log-transformed expressions of each featured gene distributed across all the seven clusters. EL, epithelial-like cells; M/m, macrophage/microglia; P, pituicyte; TL, T-cell like; VE, vascular endothelia.

10.1523/ENEURO.0345-19.2019.f1-3 Extended Data Figure 1-3 The filtered list of normalized differentially expressed genes. List of normalized differentially expressed genes in each cell type displaying an average log_2_ fold change ≥ 1 and adjusted *p* ≤ 0.05. Download Figure 1-3, XLSX file.

**Figure 4.**
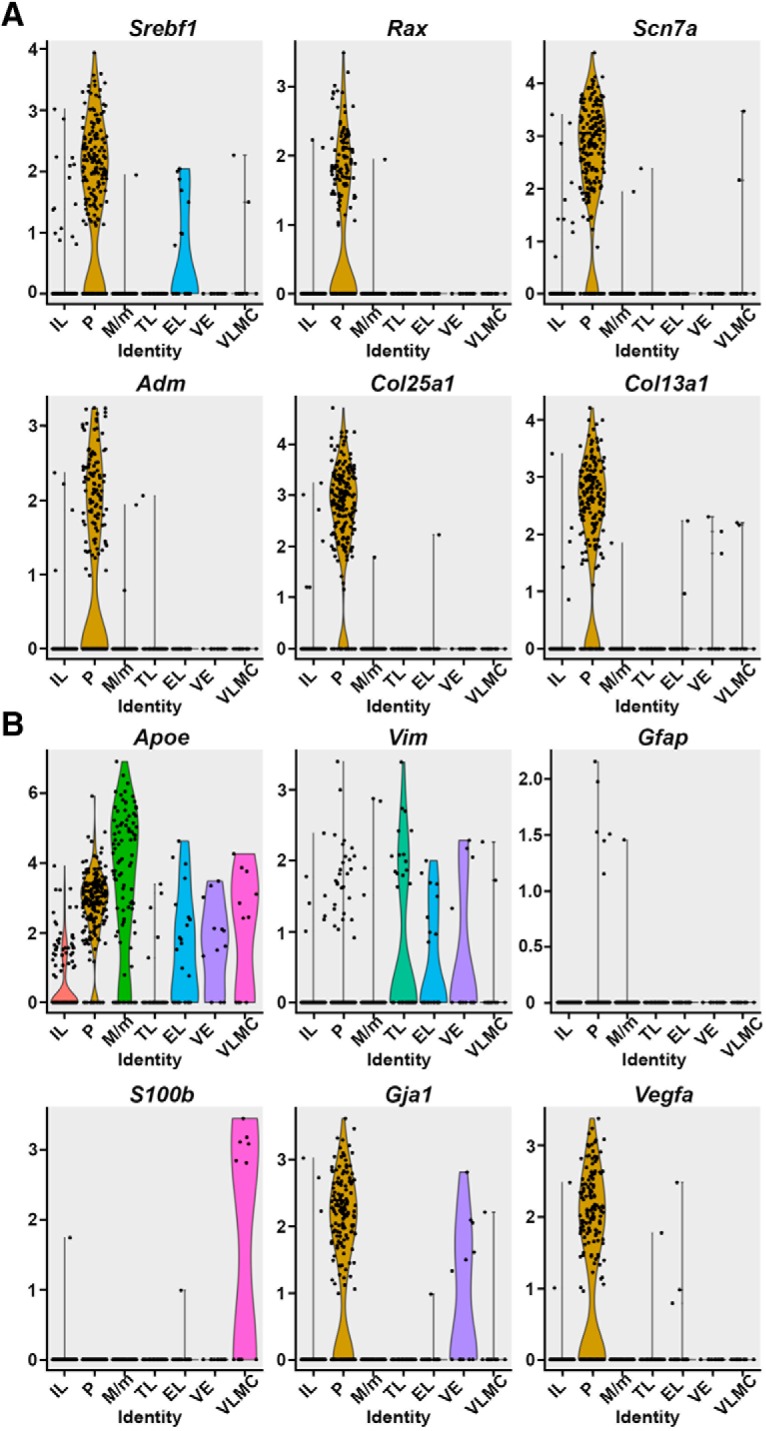
Novel pituicyte markers show higher specificity and robustness compared to previously used markers. ***A***, Violin plots displaying expression distributions of novel pituicyte marker genes in seven pituitary cell types seven clusters. *Srebf1*, *Rax*, *Scn7a*, *Adm*, *Col25a1*, and *Col13a1* were selected from this single-cell RNA-Seq data and mapped onto the violin plots. The *y*-axis represents the normalized log-transformed expression of respective genes. Each dot represents a cell and the shape of the violin represents the proportion of cells being enriched compared to the rest of cells in a given cluster. ***B***, Previously published pituicyte markers *Apoe*, *Vim*, *Gfap*, *S100β*, *Gja1 (Cx43)*, and *Vegfa* were mapped onto the violin plots within the seven identified cell types. EL, epithelial-like cells; M/m, macrophage/microglia; P, pituicyte; TL, T-cell like; VE, vascular endothelia.

10.1523/ENEURO.0345-19.2019.f1-4 Extended Data Figure 1-4 ORA of pituicyte transcriptome to PanglaoDB. A comparison of the pituicyte transcriptome to the PanglaoDB database of mouse scRNA-Seq. The list was ranked by adjusted *p* value (FDR) smallest to largest. Download Figure 1-4, XLSX file.

**Figure 5.**
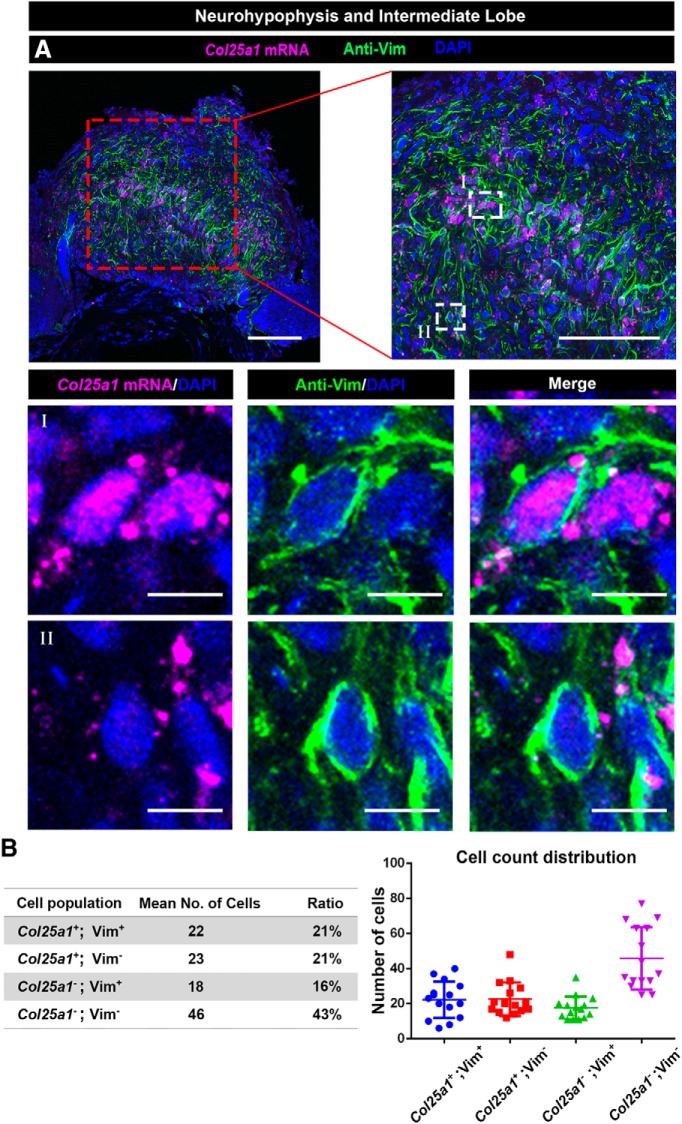
Expression of the novel pituicyte marker *Col25a1*, in the NH. ***A***, Validation of the scRNA-Seq results using wholemount staining of dissected NH derived from a C57/BL6 adult mouse. Dissected NH was subjected to fluorescent mRNA *in situ* hybridization with an antisense *Col25a1* probe, followed by immunostaining with an antibody directed to the Vim protein and visualized by confocal microscopy. The top panels display different magnifications (scale bars, 100 µm) a single confocal optical plane of *Col25a1*, *Vim*, and the nuclei dye, DAPI. Highly magnified field (scale bars, 10 µm) of views showing a representative *Col25a1****^+^****;* Vim***^+^*** pituicyte (I) and another *Col25a1****^-^****;* Vim***^+^*** neurohypophyseal cell (II). ***B***, Numbers of different subpopulation of cell expressing *Col25a1* and/or Vim were analyzed in 15 randomly chosen areas of interest (between 18,133 and 40,429 µm^2^). The average cell numbers and ratios, as well as the individual counting in each region of interest, are presented.

10.1523/ENEURO.0345-19.2019.f1-5 Extended Data Figure 1-5 Presence of pituicyte markers in astrocyte and tanycyte cells. A comparison between selected pituicyte markers (Ave LogFC > 1, *p*adj < 0.05) revealed in this study with previously published markers of astrocyte and tanycyte. The presence (green) or absence (magenta) of pituicyte markers in astrocyte and tanycyte databases are indicated. The list of all markers used for this comparison is shown in the respective sheets together with their respective references. Download Figure 1-5, XLSX file.

We collected two pools of dissected neurohypophyseal tissue, each has been derived from five mice. Single cells from the dissociated tissue were thereafter captured using the 10x chromium gel beads in a droplet, followed by independent library preparation for each pool. The two sets of libraries were indexed and sequenced together (Fig. 1*A*). The low variation between the pools was detected in the PC analysis (PCA) plot containing the two first PCs and in the tSNE plot using Seurat R package (Butler et al., 2018). The two data sets were pooled and cell clusters were built using the 900 most variable genes using FindClusters function in Seurat package using 11 PCs with resolution 1.0 and analyzed together to create the tSNE plot (Fig. 1*B*; Extended Data 1-1*B*). The normalized differentially expressed genes of each cluster (Extended Data Figs. 1-2, 1–3) were used to identify seven major cell types, which were designated based on expression of published marker genes and following comparisons to existing single-cell database (Fig. 1*B*). Thus, we compared our gene lists to the mouse brain atlas from the Linnarsson Lab (Zeisel et al., 2018), the PanglaoDB database (Franzen et al., 2019), the cell type function from Allen Brain Atlas (http://celltypes.brain-map.org/), mouse vascular and vascular associated cell single-cell database (He et al., 2018; Vanlandewijck et al., 2018), and the DropViz web tool (Saunders et al., 2018). We also compared our data to published scRNA-Seq of anatomically adjacent tissues, such as the hypothalamus and the median eminence (Campbell et al., 2017; Chen et al., 2017; Extended Data Figs. 1-4, 1-5). The identified NH cell types were labeled as: pituicyte, macrophage/microglia, vascular endothelia, T-cell like and vascular and leptomeningeal cells (VLMCs). As expected, due to the nature of the dissection procedure mentioned above, we also identified IL cells. The latter was identified by comparing to recently published whole mouse pituitary single-cell transcriptomes (Cheung et al., 2018; Ho et al., 2018; Mayran et al., 2018). To determine the relativeness of the clustered cell types, we used the BuildClusterTree function in Seurat R package to generated dendrogram, representing a phylogenetic tree relating the “average” cell from each identity class (Fig. 1*C*). The number of cells, as well as mean number of genes and average number unique molecular identifiers (UMIs) representing each of the designated cell types are shown in Figure 1*D*. Notably, the cell number does not necessarily reflect the compositional proportion in the tissue but probably randomized sampling in single-cell capturing, varied resilience of different cell types to dissociation procedure and cell type-specific RNA stability.

Following the identification of NH and IL cell types, we searched for sets of genetic markers characterizing each cell type. We generated a heatmap showing cluster analysis of the top twenty differentially expressed genes representing the transcriptomic profile of the various NH and IL cell types and then selected three feature genes, which represent each cell type (Fig. 2). These included known markers for VLMC cells (*Ogn*, *Lum*, and *Dcn*), fenestrated vascular endothelia (*Emcn*, *Flt1*, and *Plvap*), T-cell like (*Ms4a4b* and *Cd3d*), and macrophage/microglia (*Ctss*, *C1qa*, and *Cx3cr1*) (Stan et al., 1999; Liu et al., 2001; Kindt et al., 2007; Marques et al., 2016). In the case of three of the identified cell types, epithelial-like cells, pituicytes, and IL cells, there was no published database and therefore they were designated based on the top differentially expressed markers. Thus, the epithelial cell markers *Krt18*, *Krt8*, and *Clu* were top-ranked in the so-called epithelial-like cells, and the melanotrope markers *Pomc* and *Pcsk2* were used to designate IL cells. To define the pituicyte cell type we first used *Vegfa* and *Gja1*, which were previously associated with this cell type (Yamamoto and Nagy, 1993; Furube et al., 2014). Next, we performed an unbiased bioinformatics analysis by comparing our pituicyte transcriptome to PanglaoDB, a public database for exploration of mouse and human scRNA-Seq data (Franzen et al., 2019). We employed ORA, which is a widely used approach to determine if known biological functions or gene sets are overrepresented in an experimentally-derived gene list (Boyle et al., 2004). Our unbiased comparison of the pituicyte to all PanglaoDB gene sets revealed that the pituicyte cluster is highly enriched in tanycyte (FDR *=* 1.20E-21) followed by astrocytes (FDR *=* 1.18E-07) and Bergmann glia (FDR = 5.63E-06; Extended Data Fig. 1-4). To further illustrate the above finding, we compared the specific differentially expressed pituicyte markers with other published scRNA-Seq data of tanycytes (40% shared markers) and astrocytes (12% shared markers) in addition to PanglaoDB (Campbell et al., 2017; Chen et al., 2017; Saunders et al., 2018; Zeisel et al., 2018; Franzen et al., 2019; Extended Data Fig. 1-5). Therefore, the unique differentially expressed featured genes we assigned for these cell types are novel markers. Thus, the novel markers *Lcn2*, *Cyp2f2*, and *Krt18* represented epithelial-like cells; *Pcsk2*, *Scg2*, and *Chga* marked IL cells, and finally, *Col25a1*, *Scn7a*, and *Srebf1* were selected as pituicyte panel of markers (Fig. 2). The specificity of the selected marker genes is exemplified in Figure 3 in which a featured gene from each cluster is highlighted in the tSNE plot showing distinct distributions of different cell types (Fig. 3*A*). A violin plot showing the normalized log-transformed single-cell expression of selected featured genes in the different cell types is shown in Figure 3*B*.

### Novel pituicyte genes display higher specificity than commonly used markers

We report five selected differentially expressed genes, *Srebf1*, *Rax*, *Scn7a*, *Adm*, *Col25a1*, and *Col13a1*, which showed robust expression in the majority of pituicyte population (Fig. 4*A*). Four of these genes, *Srebf1*, *Rax*, *Adm*, and *Col25a1* were robustly expressed in the pituicyte population. *Srebf1* displayed residual expression in a small number of epithelial-like cells but was not differentially expressed in this cluster (Fig. 4*A*; Extended Data Figs. 1-2 and 1-3).

We noticed that the novel pituicyte genes revealed by scRNA-Seq displayed higher specificity than previously published pituicytes markers (Cocchia, 1981; Suess and Pliška, 1981; Boyles et al., 1985; Marin et al., 1989; Yamamoto and Nagy, 1993). Thus, violin plots of our scRNA-Seq indicated that two commonly used pituicyte markers *Gfap* and *S100β* displayed low normalized log-transformed expressions in the pituicyte population. Furthermore, *Apoe*, which is often used as pituicyte and astrocyte marker displayed low cell-type specificity, as it was detected in all neurohypophyseal types except for T-cell like. The other three reported pituicyte markers *Gja1/Cx43*, *Vegfa*, and *Vim* (Marin et al., 1989; Yamamoto and Nagy, 1993; Furube et al., 2014) displayed higher normalized pituicyte expression and were somewhat more specific than *Apoe* (Fig. 4*B*). Notably, although *Vim* displayed some expression in the pituicyte cells, it did not pass the differentially expressed criteria in the pituicyte cluster when compared to other cell types (Extended Data Figs. 1-2 and 1-3).

We next examined whether the novel pituicyte markers identified by scRNA-Seq are expressed in the mouse NH by *in situ* hybridization. The selected pituicyte marker *Col25a1* (Fig. 4*A*) with robust normalized expression (adjusted *p* = 8.38E-82, average ln fold change = 1.67) was subjected to wholemount mRNA *in situ* hybridization, followed by immunostaining with an antibody against the previously published pituicyte marker *Vim.* This analysis showed that *Vim* immunoreactivity is detected in a subset of *Col25a1*-positive cells (Fig. 5; Extended Data Fig. 5-1; Movie 1). This analysis was in agreement with our scRNA-Seq bioinformatic analysis (Fig. 4), suggesting that some of the commonly used pituicyte markers also label other NH cell types.

10.1523/ENEURO.0345-19.2019.f5-1 Extended Data Figure 5-1 Expression of *Col25a1* and Vim in neurohypophyseal cells. High-resolution image showing co-localization *Col25a1* mRNA and Vim protein in neurohypophyseal cells, whose nuclei were labeled by DAPI. The cytoskeletal protein Vim is expressed in the cell circumference, while *Col25a1* mRNA is localized in the cytoplasm (scale bars, 10 µm). Download Figure 5-1, TIF file.

Movie 1. WISH of *col25a1* co-stained with Vim antibody and DAPI on dissected NH of three-month-old C57/BL6. 10.1523/ENEURO.0345-19.2019.video.1

### Spatial organization of neurohypophyseal cell types

**Figure 6.**
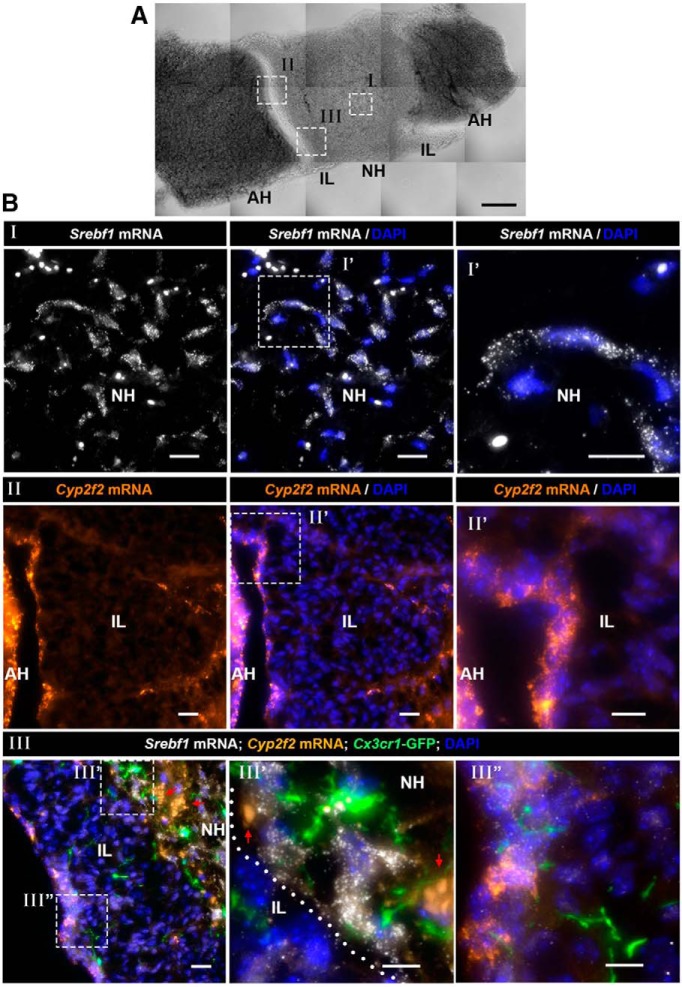
Spatial distribution of pituicyte, macrophage/microglia and epithelial-like cells in the NH and IL. ***A***, A brightfield image of a horizontal section of adult mouse pituitary showing the locations of the NH, IL, and AH. The white boxes in the brightfield image mark the locations of specific pituitary subdomains shown in the fluorescent images below (scale bar, 100 µm). ***B***, Different fields of views (marked by roman numbers) of horizontal section (7 µm) of pituitaries derived from three-month-old *Cx3cr1*-GFP macrophage/microglia transgenic reporter mouse, which were subjected to smFISH with antisense probes directed to *Srebf1* (I), *Cyp2f2* (II), or multiplexed smFISH of *Srebf1* and *Cyp2f2* on *Cx3cr1*:GFP mouse (III) to observe the relative location of selected cell types. A high-magnification image of the region delineated with the white dashed box is shown. White dotted line in III’ marks the boundary between IL and NH. Note that the smFISH probe of epithelial-like cell marker, *Cyp2f2*, labels the border between the IL and the AH, as well as IL cells. Arrows indicate background autofluorescent signals of circulating erythrocytes. Scale bars, 20 µm (I, II) and 10 µm (III).

**Figure 7.**
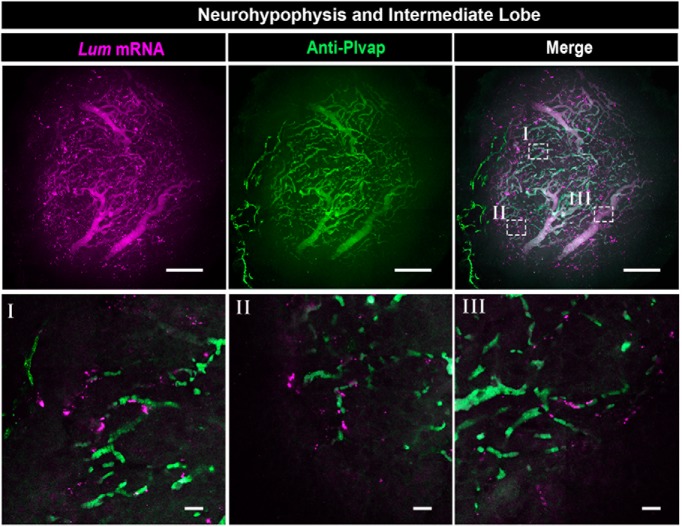
Neurohypophyseal VLMCs are associated with fenestrated vascular endothelia. Confocal Z-stack (maximum intensity projection) of dissected NH, which was subjected to wholemount FISH with an antisense RNA probe directed to the VLMC marker, *Lum*, followed by immunostaining with an antibody directed to *Plvap* protein, which is a marker of fenestrated endothelia (scale bars, 100 µm). The bottom panels (labeled I–III) display high-magnification single plane confocal images of the respective regions delineated in white boxes in the top right panel (scale bars, 20 µm).

To better understand the spatial organization of neurohypophyseal cell types, we analyzed the expression of selected genetic markers representing the major NH cell types and localized the expression on a horizontal section of whole mouse pituitary (Fig. 6*A*). We performed single molecule smFISH on a pituitary derived from a transgenic macrophage/microglia reporter (Fig. 6) as well as wholemount mRNA *in situ* hybridization combined with antibody staining (Fig. 7). Our scRNA-Seq analysis indicated that *Srebf1* is a novel pituicyte marker displaying limited expression in the epithelial-like cells, while *Cyp2f2* was highly expressed in epithelial-like cells (Figs. 3, 6*B*). Accordingly, *Srebf1* was prominently expressed in the NH (Fig. 6*B*; Extended Data Figs. 6-1, 6-2*B*), while *Cyp2f2-*expressing cells were mostly located at the boundary between the IL and the AH (Fig. 6*B*; Extended Data Fig. 6-1). Notably, *Cyp2f2* mRNA signals were much weaker in the NH compared to the IL and the AH boundary suggesting that some epithelial-like cells are also found in the NH (Fig. 6*B*; Extended Data Figs. 6-1, 6-2*B*). This conclusion was further confirmed using smFISH to probe another specific epithelial-like featured gene, *Lcn2,* which was mainly expressed by cells located at the IL and AH boundary (Extended Data Fig. 6-2*B*).

10.1523/ENEURO.0345-19.2019.f6-1 Extended Data Figure 6-1 Separate channels for multiplex smFISH images in Figure 6III’,III”. High-magnification images of the horizontal pituitary section (7 µm) shown in Figure 6BIII’,III”. The different panels show separate fluorescent emission channels derived from an adult *Cx3cr1*-GFP microglia/macrophage transgenic reporter mouse, which was subjected to smFISH with antisense probes directed to pituicyte epithelial-like cells markers *Srebf1* and *Cyp2f2*, respectively. White dotted lines indicate the boundary between IL and NH. Arrows pointing at co-localization of *Cyp2f2* mRNA and *Srebf1* mRNA in the cells at the boundary between IL and AH (scale bars, 10 µm). Download Figure 6-1, TIF file.

10.1523/ENEURO.0345-19.2019.f6-2 Extended Data Figure 6-2 Gene expression of the neurohypophyseal and IL. ***A***, A brightfield image of a horizontal section of adult mouse pituitary showing the locations of the NH, IL, and AH. The white boxes in the brightfield image mark the locations of specific pituitary subdomains shown in the fluorescent images below (scale bar, 100 µm). ***B***, Different fields of view (marked by roman numbers) of horizontal section (7 µm) of pituitaries derived from three-month-old Cx3cr1-GFP macrophage/microglia transgenic reporter mouse, which were subjected to smFISH. The separate image panels show the expression of the pituicyte marker *Srebf1* and epithelial-like marker *Cyp2f2* in the NH (I) and the epithelial-like marker *Lcn2* at the boundary of IL and AH (II), also marked by the white dotted line (scale bars, 20 µm). ***C***, Confocal Z-stack (maximum intensity projection) of horizontal vibratome pituitary section (50 µm) from a three-month-old male mouse harboring the transgenic macrophage/microglia reporter *Cx3cr1*-GFP (scale bar, 200 µm). ***D***, Immunostaining of the T-cell marker, *Cd3*, showing a lone T-cell inside the NH horizontal pituitary cryosection (7 µm) of a three-month-old C57/BL6 male mouse (scale bars, 20 µm). Download Figure 6-2, TIF file.

We next performed simultaneous labeling of macrophage/microglia, pituicyte, and epithelial-like cells by performing smFISH of *Srebf1* and *Cyp2f2* probes on pituitaries of transgenic *Cx3cr1*:GFP reporter mice, labeling macrophage/microglia (Jung et al., 2000). We observed that the *Cx3cr1*: GFP-positive macrophage/microglia were distributed throughout the whole pituitary, including the NH, IL, and AH (Fig. 6*B*; Extended Data Fig. 6-2*C*). These macrophages/microglia were intermingled with both *Srebf1****^+^****; Cyp2f2****^-^*** pituicytes and *Cyp2f2****^+^*** epithelial-like cells suggesting a possible cross-talk between pituitary cells and these macrophages/microglia (Fig. 6*B*).

Our scRNA-Seq analysis also detected an NH cell population, which co-expressed *Pdgfra* and *Lum* (Fig. 2; Extended Data Fig. 1-3). We assumed that this cell population is similar or identical to the so-called VLMC, which has been found to localized on blood vessels of the brain (Marques et al., 2016; He et al., 2018; Vanlandewijck et al., 2018). We, therefore, examined the tissue distribution of VLMC cells and fenestrated neurohypophyseal vascular endothelia, which express the *Plvap* protein (Stan et al., 1999; Gordon et al., 2019). This analysis confirmed that as in the case of brain vasculature, VLMCs were in close association with the fenestrated endothelia of the NH (Fig. 7). Finally, although we have found a small population of T-cell like cells in the NH, immunostaining of the T-cell-specific cell surface marker, *Cd3*, revealed low abundance of *Cd3-*positive cells in the NH (Kindt et al., 2007; Extended Data Fig. 6-2*D*). It is likely that these T cells are not a resident NH population but rather a transient population, which is transported from the blood. The above *in situ* hybridization analyses confirmed our featured gene designation determined by scRNA-Seq.

Taken together, our gene expression analysis of NH and IL reveals a comprehensive view of neuro-IL cell types in adult male mice. This study provides an important resource for specific functional studies and possible crosstalk between the various NH cell types.

## Discussion

The NH is a major neuroendocrine interface, which allows the brain to regulate the function of peripheral organs in response to specific physiologic demands. Despite its importance, a comprehensive molecular description of cell identities in the NH is still lacking. Recent studies revealed cell heterogeneity of whole pituitary gland using scRNA-Seq, however, these studies did not separate the NH from the adjacent AH and very few NH cells with limited sequence information were reported (Cheung et al., 2018; Ho et al., 2018; Mayran et al., 2018). Here, utilizing scRNA-Seq technology, we identified the transcriptomes of five major neurohypophyseal cell types and two IL cell populations in the adult male mice. Using selected featured genetic markers, we mapped the spatial distribution of selected cell types *in situ*.

The identified differentially expressed gene clusters revealed by scRNA-Seq correspond to previously characterized cell types. Thus, previous studies reported the appearance of pituicyte (Seyama et al., 1980; Wittkowski, 1986; Yamamoto and Nagy, 1993; Furube et al., 2014; Anbalagan et al., 2018), macrophage/microglia (Pow et al., 1989; Sasaki and Nakazato, 1992; Kindt et al., 2007), and fenestrated endothelia (Gordon et al., 2019) in the NH. The identification of IL and epithelial-like cells in the present study is in agreement with other reports of these cells in the mammalian pituitary (Hudson, 2002; Moran et al., 2011) and also matches recent scRNA-Seq analyses of whole mouse pituitary (Cheung et al., 2018; Ho et al., 2018; Mayran et al., 2018).

The novel pituicyte markers identified in our study showed more specific and robust expression than previously published pituicyte markers. Among them, *Srebf1*, *Col13a1*, *Adm*, *Scn7a*, and *Col25a1* were not reported to be expressed by pituicyte. *Vegfa* was reported as pituicyte marker in both mice and zebrafish (Furube et al., 2014; Anbalagan et al., 2018). Srebf1 protein is involved in sterol biosynthesis process, this may be relevant to the lipid droplets that were found in ultra-structure studies of pituicyte (Seyama et al., 1980; Wittkowski, 1986; Anbalagan et al., 2018). Other prominent pituicyte markers we identified, such as *Rax*, *Scn7a*, *Col25a1*, and *Adm* were reported as hypothalamic tanycyte markers (Miranda-Angulo et al., 2014; Pak et al., 2014; Campbell et al., 2017; Chen et al., 2017; Franzen et al., 2019). Our finding that *Rax*, *Scn7a*, *Col25a1*, and *Adm* are expressed in pituicytes is in line with the notion that tanycytes and pituicytes are of a common astrocytic lineage (Wittkowski, 1998; Clasadonte and Prevot, 2018; Rodríguez et al., 2019). Specifically, *Rax* is a general tanycyte marker (Campbell et al., 2017; Chen et al., 2017; Rodríguez-Rodríguez et al., 2019), *Scn7a*, *Col25a1*, and *Adm* were reported as β2 tanycyte markers (Campbell et al., 2017; Rodríguez-Rodríguez et al., 2019). Finally, *Col25a1* was found to be enriched in the NH according to the Bgee database (Bastian et al., 2008).

Our novel pituicyte markers displayed greater specificity (i.e., adjusted *p* ≤ 0.05 for differential expression), higher expression level (average ln fold change ≥ 1) and robustness (i.e., abundance in pituicytes) compared to the most commonly used markers. Thus, as we previously showed in the case of zebrafish pituicytes (Anbalagan et al., 2018), we found that *Apoe* is broadly expressed in multiple mouse NH cell types. However, although *Vim* and *Gfap* displayed relatively low mRNA expression levels in our scRNA-Seq analysis, their protein immunoreactivity was readily detectable in the NH. This could be due to the inherently shallow sequencing method for 10x Genomics platform. The astroglial protein *S100β* is also used to label pituicytes (Cocchia, 1981). It was reported that *S100β* is highly abundant when compared to *Vim^+^* and *Gfap^+^* cells (Virard et al., 2008; Wei et al., 2009). However, in our study, *S100β* was not among the top differentially-expressed pituicyte genes but was found to be exclusively expressed in the VLMC cell type. In view of the gene coverage limitation of the 10x Genomics platform, *S100β* might have been missed in our analysis, hence, future studies should be aware of our findings regarding its expression in VLMC. Another known pituicyte-specific marker, namely *Gja1*, also known as *Cx43*, displayed robust specific expression in our mouse pituicyte cluster. This is in agreement with the reported findings in rat and zebrafish (Yamamoto and Nagy, 1993; Anbalagan et al., 2018).

We identified VLMC as a new neurohypophyseal cell type which is marked by the prominent expression of *Pdgfra* and *Lum.* We further showed that VLMC is associated with *Plvap^+^* fenestrated neurohypophyseal capillaries. In agreement with our findings, *Pdgfra^+^*;*Lum* ^+^ VLMC population was found in the mouse brain as vascular-associated cell type (Marques et al., 2016) or as fibroblast-like cells that are loosely attached to vessels and located in between smooth muscle cells and astrocyte end-feet (Vanlandewijck et al., 2018). Although VLMC express some markers of oligodendrocyte precursor cells (OPCs), such as *Pdgfra*, they are distinct from OPCs and oligodendrocyte lineages (Marques et al., 2016).

Importantly, previous reports have described the existence of OPCs in the NH (Virard et al., 2006, 2008; Miyata, 2017). We did not detect OPCs in the present study, however, this could be due to the low abundance of these cells in our tissue. Alternatively, because Virard et al. relied on *Pdgfra* as a sole OPC marker, it is possible that they misidentified VLMCs as OPCs. Notably, Virard et al. reported that these *Pdgfra* ^+^ cells were shown to be pituicyte progenitors in their study (Virard et al., 2006). Similarly, other studies reported that VLMC display multipotent stem cell niche function in the CNS and other organs suggesting that they may play similar roles in NH function (Nakagomi and Matsuyama, 2017; Ueharu et al., 2018). Further studies are required to determine whether neurohypophyseal OPCs are in fact VLMCs and whether VLMCs are pituicyte progenitors.

Although pericytes have been previously reported to be associated with neurohypophyseal capillaries (Miyata, 2017; Nishikawa et al., 2017), we did not detect them in the present study, possibly due to the fact that isolating pericytes requires different tissue dissociation conditions. It is also possible that other minor neurohypophyseal cell populations have been missed, which may be revealed if more cells would be sampled.

Macrophage/microglia were found in our study as prominent NH resident cells. Previous reports showed that neurohypophyseal microglia in rat endocytose and digest axonal terminus (Pow et al., 1989), whereas the pituicyte envelops the buttons of axons (Morris, 1976) and provide cues for the permeable endothelial fate (Anbalagan et al., 2018). Our finding that macrophage/microglia are closely located to the pituicytes in the NH is in agreement with such functional cooperation between these two cell types.

In summary, our transcriptome analysis of individual cells derived from NH and IL tissues of adult male mice have revealed the cellular heterogenicity of the NH and provide novel molecular markers for the major cells in those tissues. We present a valuable resource that will serve as the basis for further functional studies.

## Synthesis

Reviewing Editor: Quentin Pittman, University of Calgary Decisions are customarily a result of the Reviewing Editor and the peer reviewers coming together and discussing their recommendations until a consensus is reached. When revisions are invited, a fact-based synthesis statement explaining their decision and outlining what is needed to prepare a revision will be listed below. The following reviewer(s) agreed to reveal their identity: Masha Prager-Khoutorsky, Marysia Placzek. Note: If this manuscript was transferred from JNeurosci and a decision was made to accept the manuscript without peer review, a brief statement to this effect will instead be what is listed below.

The present study investigates expression profiles of different neurohypophysial (NH) cell types using single cell RNA sequencing. The main aim of the study is to identify novel and more specific cellular markers for different cell types in the area, particularly for resident NH astrocytes - pituicytes, to facilitate future studies examining their functional role. This is a very important study since specific markers of pituicytes are missing, and typically more traditional astrocytic markers have been used to distinguish these cells (e.g. GFAP, s100b), as well radial-glia markers such as vimentin. Identifying a unique marker of pituicytes will contribute not only an important research tool, but may also provide new insights into understanding of their unique function.

Reviewer 1

Major comments:

The most important finding of this study is identification of novel pituicyte markers (Col25a1, Mest and Srebf1). However, it's unclear if authors suggest that pituicytes represent a distinct cell population that is different from other astrocyte types. If authors assume that these markers are unique for pituicytes, they should provide evidence that the most closely-related glia cell type, astrocytes (outside NH), do not express these markers. Thus, additional data will be needed to confirm the presence or the absence of these markers in astrocytes (outside of NH).

Minor comments:

1. Minor editing: Some abbreviations should be defined, e.g. what is PCA (pages 9 and 24 (fig1 legend)); abbreviations are not defined first time used but later in the text, e.g. PCs. There are occasional spelling/grammar errors that should be addressed prior to publication.

2. Since authors suggest that common cell markers currently used show no differential expression in different cell populations (epithelial-like cells, pituicytes, and IL cells) (page 10: “the majority of the commonly used markers for these cell types were not differentially expressed between the cluster”. Then how the authors concluded that new set of markers represent different cell populations? Is it possible that, e.g. for pituicytes, there are several sub-populations (represented by two clusters), some of which express a novel marker (e.g. Krt18, identified by authors as epithelial marker) and others do not? The basis for the conclusion why these populations were identified as different cell types should be better explained.

3. For fig. 5, there is no co-localization between vimentin and Col25a1 mRNA, and thus it is hard to judge if the marker is really expressed in Vim-positive cells (putative pituicytes). It is conceivable, that vimentin labelling is corresponding to other cell processes wrapping around the Col25a1 mRNA-positive DAPI positive cells. Perhaps if such co-localization cannot be seen with vimentin, authors might use another glial marker that shows more clear localization to cells bodies to demonstrate colocalization with Col25a1 mRNA.

4. In fig. 5 panel I, all mRNA is co-localizing with nucleus (DAPI). Could authors provide explanations with mRNA is in the nucleus?

5. In fig 6 III, it's hard to see whether there is Screbf1 mRNA in the III' (IL region). Based on the authors' statement in results (page 12), the marker of pituicytes is not found there. But there are lots of white puncta seen in fig 6 III', which may be due to the unsuccessful pseudo-color choice. If so, the color coding should be modified to illustrate authors' conclusion that there is no pituicytes in IL and Screbf1 mRNA is missing in this area.

6. Also, the authors state that “Cyp2f2-positive cells were not found in the NH...”, however there is a clear appearance of Cyp2F2 mRNA -positive signal (putative epithelial cells) in the NH (fig 6 III'), what are these epithelial-like cells in the NH?? The authors attribute this signal to autofluorescence from red blood cells, and thus the panel doesn't look very convincing. First, the presence of red blood cells suggests the tissue wasn't well perfused, also the red blood cells fluorescence typically appears not only on red but also green channel. Taking together, the authors choose to show an image of tissue where “orange” can be also associated with Cyp2f2 signal is confusing. To be more convincing, the authors should show a better-quality image of NH showing no Cyp2f2 staining.

7. In page 12 (very end), the authors say that “Some of these Cyp2f2+ IL cells coexpressed Srebf1, however they were spatially separated from pituicytes located in the NH”. That contradicts their previous statement that Cyp2f2 is an exclusive marker for epithelial cells, e.g. earlier on the same page, its written: “Our scRNA-Seq analysis indicated that Srebf1 is a novel pituicyte marker displaying limited expression in the epithelial like cells, while Cyp2f2 was exclusively expressed in epithelial-like cells”. Please explain how to reconcile these confusing statements.

8. The conventional markers of pituicytes such as vimentin, S100B, and GFAP show a very low to none (for s100b) mRNA expression, yet have very high protein content, clearly suggesting that mRNA levels have poor prediction power with regards to the protein abundance. Is the use of these markers limited to mRNA for in situ hybridization? Does the expression of these mRNAs reflect high protein levels specifically in corresponding cell types? Based on the data, it is uncertain if the markers identified using mRNA expression can be good research tool to label corresponding proteins in pituicytes.

9. Lastly, a discussion regarding what proteins are encoded by these major cell specific mRNA would greatly improve the impact of the study.

Reviewer 2

The study is likely to be influential in the field with the discovery of novel markers of different cell types, and localised expression. A key cell type analysed is the pituicyte - a poorly-understood cell type that is critical to the brain-body interface. Importantly, the data reveals that previous 'pituicyte' markers are also expressed on other cells in the NH - but then goes on to identify novel pituicyte markers. The manuscript is therefore likely to be highly cited as future studies use this paper as a resource.

Minor comments:

1.The scRNA Seq data looks good, but I was struck that the data is based on very low cell numbers for each of the described population of cells, and a limited number of genes. I would have expected more cells from 5 adult pituitaries. The only reason this is a concern is if particular population(s) have been lost. The authors may wish to comment on this in the discussion.

2. Fig1 - either a cartoon or image of the region that has been dissected out for RNA seq would help in terms of the landmarks described in the text.

3. I could not find Table 1-1 and 1-2.

4. A detailed list of all the differentially expressed genes for each of the 7 populations would be helpful. This should include the previously published markers used, in the first instance, to classify each population.

5. The logic around identification of pituicyte is circular. The authors state that current markers are not specific enough to properly identify them, but then use these to identify this group, then attribute the newly discovered genes to a subset of these - how can the authors be sure that these are actually pituicytes? Is it just through the cluster analysis in Fig 1B? Can they be certain that this is a single population (ie a pituicyte population)

6. Similarly - Fig4 - the data is difficult to interpret, as Mest expression looks equally high in IL and P cells and Vimentin is barely detected in pituicytes; similarly why are the commonly used pituicytes markers barely expressed in these plots although the main criteria by which they are defining/designating these population is via the these published marker gene expression?

7. Fig 5: The comparison between Col25a1 and Vimentin provides evidence that current pituicyte markers also label non-pituicyte cells. However, Figure 5 would benefit with some stats to show Col25a1+ Vim+ cell number vs Col25a1-Vim+ cell number.

8. Fig 6. The suggestion that Cyp2f2 and srebf1 are co-expressed in pituicytes needs to be confirmed by showing separate channel views.

For the non-expert, Figure 6A would benefit from a complimentary cartoon to get a sense of what the pituitary looks like in the animal and where the different domains are.

10. The authors argue that macrophages and pituicytes are intermingled in the tissue and that this suggests functional cell-cell interactions between these cell types - this is too strong a conclusion.

## Author Response

oint-by-point responses to the reviewers' comments to

Chen et al. MS ID: eN-TNWR-0345-19X

Reviewer 1 Major comments:

The most important finding of this study is identification of novel pituicyte markers (Col25a1, Mest and Srebf1). However, it's unclear if authors suggest that pituicytes represent a distinct cell population that is different from other astrocyte types. If authors assume that these markers are unique for pituicytes, they should provide evidence that the most closely-related glia cell type, astrocytes (outside NH), do not express these markers. Thus, additional data will be needed to confirm the presence or the absence of these markers in astrocytes (outside of NH).

As requested, we have now established that pituicytes represent a distinct cell population that is different from other astrocyte types: 1) As an unbiased bioinformatics analysis, we compared our pituicyte transcriptome to PanglaoDB, a public database for exploration of mouse and human single-cell RNA sequencing data (Franzen et al., 2019). We employed an Over Representation Analysis (ORA) (Boyle et al., 2004), which is a widely used approach to determine whether known biological functions or gene sets are over-represented (enriched) in an experimentally-derived gene list (see 'Materials&Methods' for details). Our unbiased comparison of the pituicyte to all PanglaoDB gene sets revealed that the pituicyte cluster is highly enriched in tanycyte (FDR=1.20E-21) followed by astrocytes (FDR=1.18E-07) and Bergmann glia (FDR=5.63E-06) (See new Table 1-3).

2) To further illustrate the above finding we prepared a new Table (Table 1-4) in which we compared the specific differentially expressed pituicyte markers with other published single-cell RNA sequencing data of tanycytes (40% shared markers) and astrocytes (12% shared markers) in addition to PanglaoDB (Campbell et al., 2017; Chen et al., 2017; Franzen et al., 2019; Saunders et al., 2018; Zeisel et al., 2018) (See new Table 1-4). These new results are now described in the revised manuscript (page 10, line 224).

Minor comments:

1. Minor editing: Some abbreviations should be defined, e.g. what is PCA (pages 9 and 24 (fig1 legend)); abbreviations are not defined first time used but later in the text, e.g. PCs. There are occasional spelling/grammar errors that should be addressed prior to publication.

We thank the reviewer for pointing this. The abbreviations and well as spelling errors have been rectified.

2. Since authors suggest that common cell markers currently used show no differential expression in different cell populations (epithelial-like cells, pituicytes, and IL cells) (page 10: “the majority of the commonly used markers for these cell types were not differentially expressed between the cluster”. Then how the authors concluded that new set of markers represent different cell populations? Is it possible that, e.g. for pituicytes, there are several sub-populations (represented by two clusters), some of which express a novel marker (e.g. Krt18, identified by authors as epithelial marker) and others do not? The basis for the conclusion why these populations were identified as different cell types should be better explained.

2

As the reviewer suggested, the method of cell type identification is now better explained in the revised manuscript (page 7 line 135). In short, the initial gene clustering was based on unbiased bioinformatics analysis of the normalized differentially expressed genes of each cluster. The subsequent cell-type designation was based on comparing our gene lists to the existing single-cell database, such as the mouse brain atlas from the Linnarsson Lab (Zeisel et al., 2018) and other databases that are detailed in our manuscript. As we indicated in the response to the major comment of this reviewer, the pituicyte cluster is enriched with known astrocyte and tanycyte markers (see new Tables 1-3 and 1-4). We further determine the phylogenetic relationship between the identified cell types. The latter analysis clearly indicates that the epithelial-like cell population is a distinct cell type (Figure 1C). This is further supported by our results showing that cells expressing featured marker genes of the epithelial cells (i.e. Lcn2 and Cyp2f2) and are primarily located in the intermediate lobe and adenohypophysis but not in the neurohypophysis (Figure 6 and Figures 6-1 and 6-2B).

3. For fig. 5, there is no co-localization between vimentin and Col25a1 mRNA, and thus it is hard to judge if the marker is really expressed in Vim-positive cells (putative pituicytes). It is conceivable, that vimentin labelling is corresponding to other cell processes wrapping around the Col25a1 mRNA-positive DAPI positive cells. Perhaps if such co-localization cannot be seen with vimentin, authors might use another glial marker that shows more clear localization to cells bodies to demonstrate colocalization with Col25a1 mRNA.

As the reviewer pointed out, Vimentin is a cytoskeletal component and its subcellular distribution is different than the cytoplasmic localized Col25a1 mRNA. The magnified fields of views in Figure 5 clearly show that Vimentin is expressed in the cell circumference and that there are two types of NH cell populations: one that co-express Vimentin and Col25a1, and another in which the two markers do not colocalize. To better demonstrate this, we added to the revised manuscript, another representative example of Vimentin and Col25a1 co-localization (new Figure 5-1A). 4. In fig. 5 panel I, all mRNA is co-localizing with nucleus (DAPI). Could authors provide explanations with mRNA is in the nucleus?

The apparent nuclei staining of Col25a1 mRNA is in fact localized in the cytoplasm around and above the nuclei of the pituicyte which occupies a relatively large portion of this cell type. This can be clearly seen by looking at a different confocal optical section which has now added as a supplementary figure (new Figure 5-1A).

5. In fig 6 III, it's hard to see whether there is Screbf1 mRNA in the III' (IL region). Based on the authors' statement in results (page 12), the marker of pituicytes is not found there. But there are lots of white puncta seen in fig 6 III', which may be due to the unsuccessful pseudo-color choice. If so, the color coding should be modified to illustrate authors' conclusion that there is no pituicytes in IL and Screbf1 mRNA is missing in this area.

The reviewer is correct and Fig. 6 indeed shows that some IL cells weakly express Srebf1 (Figure 6-1). This is also exemplified in the tSNE plots (Figure 3A) and the violin plots (Figures 3B and 4A); both show that Srebf1 is mainly expressed in pituicytes but also in some of the epithelial-like cells. This emphasizes the notion, which is demonstrated in our manuscript, that a single marker is not sufficient to define a cell type and the intersect of multiple markers is required.

6. Also, the authors state that “Cyp2f2-positive cells were not found in the NH...”, however there is a clear appearance of Cyp2F2 mRNA -positive signal (putative epithelial cells) in the NH (fig 6 III'),

3

what are these epithelial-like cells in the NH?? The authors attribute this signal to autofluorescence from red blood cells, and thus the panel doesn't look very convincing. First, the presence of red blood cells suggests the tissue wasn't well perfused, also the red blood cells fluorescence typically appears not only on red but also green channel. Taking together, the authors choose to show an image of tissue where “orange” can be also associated with Cyp2f2 signal is confusing. To be more convincing, the authors should show a better-quality image of NH showing no Cyp2f2 staining.

The reviewer is correct that the red blood cell should be also detected in the green channel. The image we presented was from a lower intensity exposure due to the fact that Cx3cr1-GFP signal is very intense compared to the Cy5 and TMR channels. A higher intensity image in which red blood cells are detectable in the GFP channel (red arrowheads) is shown below together with TMR and Cy5 channel showing Cyp2f2 and Srebf1 mRNA, respectively (Scale bars, 10µm). Having said that, the reviewer is correct that cells expressing very low level of Cyp2f2 are also found in the neurohypophysis (Figure 6-2B), once again demonstrating the notion that the intersect of multiple markers is required to define a cell type.

7. In page 12 (very end), the authors say that “Some of these Cyp2f2+ IL cells coexpressed Srebf1, however they were spatially separated from pituicytes located in the NH”. That contradicts their previous statement that Cyp2f2 is an exclusive marker for epithelial cells, e.g. earlier on the same page, its written: “Our scRNA-Seq analysis indicated that Srebf1 is a novel pituicyte marker displaying limited expression in the epithelial like cells, while Cyp2f2 was exclusively expressed in epithelial-like cells”. Please explain how to reconcile these confusing statements.

In view of the reviewer's comment, we removed the word “exclusively”. We also agree that the above sentences are somewhat confusing. To clarify, Cyp2f2 is expressed in the epithelial-like cell type (Figure 3A, B) and according to our smFISH analysis, these epithelial-like cells reside in the IL (Figure 6B, Figure 6-1 and Figure 6-2B). A subset of these epithelial-like cells co-express Cyp2f2 and Srebf1 (Figure 6-1). This is also in agreement with our tSNE analysis and violin plots (Figures 3 and 4). We revised the relevant statements to sharpen the message (page 13, line 282).

8. The conventional markers of pituicytes such as vimentin, S100B, and GFAP show a very low to none (for s100b) mRNA expression, yet have very high protein content, clearly suggesting that mRNA levels have poor prediction power with regards to the protein abundance. Is the use of these markers limited to mRNA for in situ hybridization? Does the expression of these mRNAs reflect high protein levels specifically in corresponding cell types? Based on the data, it is uncertain if the markers identified using mRNA expression can be good research tool to label corresponding proteins in pituicytes.

4

The fact that mRNA levels do not necessarily represent the corresponding protein levels is indeed a valid point for every transcriptomics analysis. However, in our hands, GFAP protein expression is not high in the neurohypophysis except in the dorsal neurohypophyseal part (see below, scale bar, 100µm). As the reviewer noticed, this finding is also in agreement with our single-cell transcriptomics.

9. Lastly, a discussion regarding what proteins are encoded by these major cell specific mRNA would greatly improve the impact of the study.

We added a description of the nature of the relevant proteins in the revised text (page 15 line 330).

5

Reviewer 2

Minor comments:

1.The scRNA Seq data looks good, but I was struck that the data is based on very low cell numbers for each of the described population of cells, and a limited number of genes. I would have expected more cells from 5 adult pituitaries. The only reason this is a concern is if particular population(s) have been lost. The authors may wish to comment on this in the discussion.

The reviewer is correct that the number of cells is low. We analyzed two independent pools of dissected neurohypophysis with a targeted recovery of 4000 cells per sample. However, after some quality checks and bioinformatics data filtration, we recovered high-quality information from a smaller portion of the loaded cells. We reason that the dissociated cells from dissected neurohypophyseal tissue are more sensitive to the 10xGenomics single-cell droplet procedure. We encountered a similar problem with zebrafish neurohypophyseal single-cell analysis (not shown). The relatively low gene number is a function of the high statistical stringency we employed in the subsequent bioinformatics analyses; however, the complete raw dataset was deposited to a public repository [Gene Expression Omnibus (GEO) (www.ncbi.nlm.nih.gov/geo): GSE135704] which is available for anyone who wishes to re-analyze our data. We agree with the reviewer that it is possible that a minor neurohypophyseal population might have been lost and this is now mentioned in the revised manuscript (page 17 line 374).

2. Fig1 - either a cartoon or image of the region that has been dissected out for RNA seq would help in terms of the landmarks described in the text.

To better illustrate the anatomy of the mouse pituitary we now present a new image of the region of interest before and after the neurohypophysis has been dissected (Figure 1-1A).

3. I could not find Table 1-1 and 1-2.

Table 1-1and 1-2 are supplemental Microsoft Excel files which are accessible to download from the journal. They are also accessible through GEO record GSE135704 (www.ncbi.nlm.nih.gov/geo) using the following secure token: uxqtuiggrfkxpkf

4. A detailed list of all the differentially expressed genes for each of the 7 populations would be helpful. This should include the previously published markers used, in the first instance, to classify each population.

This information was also made available in Table 1-1 and 1-2, which appears as supplemental files online. Also, see the new tables comparing pituicytes to published scRNA-Seq markers for astrocytes and tanycyte (Tables 1-3 and 1-4).

5. The logic around identification of pituicyte is circular. The authors state that current markers are not specific enough to properly identify them, but then use these to identify this group, then attribute the newly discovered genes to a subset of these - how can the authors be sure that these are actually pituicytes? Is it just through the cluster analysis in Fig 1B? Can they be certain that this is a single population (ie a pituicyte population)

It is well accepted that the pituicyte are specialized astroglia and therefore their cell-type designation was initially based on comparing our gene lists to the existing single-cell database, such as the mouse brain atlas from the Linnarsson Lab (Zeisel et al., 2018) and other databases that are

6

detailed in our manuscript. In response to this comment and a similar comment by Reviewer #1 (see above), we present a comparison between the complete list of the differentially expressed pituicyte markers to publish single-cell sequencing data of astrocytes and tanycytes (New Tables 1-3 and 1-4). We found that as expected, some of the pituicyte markers revealed in our study are shared between astrocytes and pituicytes, while other markers are uniquely expressed in pituicytes. Moreover, as previously suggested (Clasadonte and Prevot, 2018; Wittkowski, 1998), we show that our pituicyte transcriptome is closely related to that of tanycytes. As the reviewer indicated, we cannot overrule the possibility that there are sub-types of pituicytes that may be revealed in future studies.

6. Similarly - Fig4 - the data is difficult to interpret, as Mest expression looks equally high in IL and P cells and Vimentin is barely detected in pituicytes; similarly why are the commonly used pituicytes markers barely expressed in these plots although the main criteria by which they are defining/designating these population is via the these published marker gene expression?

We agree with the reviewer that in view of the high expression of Mest in IL cells, it is not an ideal feature gene for the pituicyte cell. Therefore, we now omitted Mest from the violin plot in Fig. 4 and instead show Scn7a and Col13a1 as pituicyte marker genes. As we mentioned in our response to Reviewer #1, we do not rely on a single gene for the designation of a cell type. We base our cell-type classification on whether a group of differentially expressed genes are enriched in previously known cell type markers (Tables 1-3 and 1-4).

7. Fig 5: The comparison between Col25a1 and Vimentin provides evidence that current pituicyte markers also label non-pituicyte cells. However, Figure 5 would benefit with some stats to show Col25a1+ Vim+ cell number vs Col25a1-Vim+ cell number.

As requested, we added the quantification of the proportion of Col25a1+; Vim+, Col25a1+; Vim-, Col25a1-; Vim+ cells and Col25a1-; Vim- cells (New Figure 5B). In short, we randomly selected 15 fields of view (the area between 18133 to 40429 µm^2, each), and calculated the proportions of the above cell populations.

8. Fig 6. The suggestion that Cyp2f2 and srebf1 are co-expressed in pituicytes needs to be confirmed by showing separate channel views.

As requested, separate channel views of Cyp2f2 and Srebf1 mRNA are shown in Fig. 6-1 with arrows added indicating co-expression.

For the non-expert, Figure 6A would benefit from a complimentary cartoon to get a sense of what the pituitary looks like in the animal and where the different domains are.

As mentioned above we have added a new image of the mouse pituitary and the neurohypophysis within it before and after the dissection procedure (Figure 1-1A).

10. The authors argue that macrophages and pituicytes are intermingled in the tissue and that this suggests functional cell-cell interactions between these cell types - this is too strong a conclusion.

The reviewer is correct. We now toned down our statement regarding the functional interaction
